# Cervical and Ocular Vestibular-Evoked Myogenic Potentials in Patients With Intracochlear Schwannomas

**DOI:** 10.3389/fneur.2020.549817

**Published:** 2020-10-27

**Authors:** Laura Fröhlich, Ian S. Curthoys, Sabrina Kösling, Dominik Obrist, Torsten Rahne, Stefan K. Plontke

**Affiliations:** ^1^Department of Otorhinolaryngology, Head and Neck Surgery, Martin Luther University Halle-Wittenberg, Halle (Saale), Germany; ^2^Vestibular Research Laboratory, School of Psychology, The University of Sydney, Sydney, NSW, Australia; ^3^Department of Radiology, Martin Luther University Halle-Wittenberg, Halle (Saale), Germany; ^4^ARTORG Center for Biomedical Engineering Research, University of Bern, Bern, Switzerland

**Keywords:** third window, vestibular schwannoma, intralabyrinthine, intracochlear, VEMP, asymmetry, secondary hydrops, semicircular canal dehiscence

## Abstract

**Objective:** To evaluate ocular and cervical vestibular evoked myogenic potentials (oVEMPs and cVEMPs) in patients with solely intracochlear localization of an intralabyrinthine schwannoma (ILS).

**Study Design:** Retrospective analysis of a series of cases.

**Setting:** Monocentric study at a tertiary referral center.

**Patients:** Patients with intracochlear schwannoma (ICS) and VEMP measurements.

**Outcome Measures:** Signed asymmetry ratio (AR) of cVEMPs and oVEMPs to air conducted sound with AR cut-offs considered to be asymmetrical when exceeding ±30% for cVEMPs and ±40% for oVEMPs with respect to the side affected by the tumor (reduced amplitudes on the affected side indicated by negative values, enhanced amplitudes by positive values); VEMP amplitudes and latencies; tumor localization in the cochlear turn and scala.

**Results:** Nineteen patients with a solely intracochlear tumor (ICS patients) [10 males, 9 females, mean age 57.1 (SD: 13.4) years] were included in the study. On the affected side, cVEMPs were absent or reduced in 47% of the patients, normal in 32%, and enhanced in 21%. Ocular VEMPs on the affected side were absent or reduced in 53% of the patients, normal in 32% and enhanced in 15%. Latencies for cVEMPs and oVEMPs were not significantly different between the affected and non-affected side. In all patients with enhanced VEMPs, the tumor was located in the scala tympani and scala vestibuli.

**Conclusions:** As a new and unexpected finding, VEMP amplitudes can be enhanced in patients with intracochlear schwannoma, mimicking the third window syndrome.

## Introduction

It was observed that intralabyrinthine schwannomas (ILS) can mimic various common cochleovestibular diseases in their symptoms and findings in functional tests. Cochleovestibular schwannomas in general, often also referred to as vestibular schwannomas or acoustic neuromas, are benign tumors that arise from the Schwann cells of the eighth cranial nerve. The schwannomas are referred to as ILS, when they arise from the most peripheral branches of the cochlear or vestibular nerves, i.e., inside the membranous labyrinth (1). ILS can present e.g., with sudden, progressive, or fluctuating hearing loss, pseudo-conductive hearing loss, and/or vertigo, and/or (pulsating) tinnitus, and have been misdiagnosed e.g., as Menière's disease (MD) or sudden hearing loss (2–8). The diagnosis is based on high-resolution magnetic resonance imaging (MRI). Various slightly differing classifications of these tumors have been suggested in the literature (2, 5, 9). The most recent and detailed classification was suggested by Van Abel et al. (5) distinguishing intracochlear, intravestibular, intravestibulocochlear, transmodiolar, transmacular, tympanolabyrinthine, translabyrinthine, and transotic locations or extensions. An extension from the internal auditory canal into the cerebellopontine angle is possible. Van Abel et al. (5) have also described that vertigo and imbalance were commonly reported when the tumors also extended to the vestibular labyrinth but were only reported by 36% of patients with intracochlear schwannomas (ICS). Intracochlear tumor localization seems to be the most common in ILS (2, 9).

The recording of cervical and ocular vestibular evoked myogenic potentials (cVEMPs, oVEMPs) has been described as a screening tool for the assessment of nerve of origin in patients with cochleovestibular schwannoma (10). However, only few data are available on VEMPs in patients with an ILS. Lee et al. (11) described absent or decreased cVEMPs and oVEMPs in patients with ILS without specifying the exact tumor location. Ralli et al. (12) reported an absent oVEMP in a patient with an intravestibular ILS with presence of “a solid mass in the utricle” confirmed by magnetic resonance imaging (MRI). Dubernard et al. (6) analyzed cVEMPs in 36 (32%) of their reported 110 patients with ILS. Twelve patients had an intracochlear tumor and cVEMPs were abnormal, i.e., absent or significantly reduced, in 50% of these patients and preserved in the remaining 50%. To date, there is no study in which both oVEMPs and cVEMPs were systematically analyzed in a series of patients with solely intracochlear tumors.

VEMPs have also been described to be highly sensitive to changes in the inner ear fluid dynamics and to detect defects of the bony labyrinthine wall. In 1998, Minor et al. (13) were the first to report about patients with a defect in the bony wall of the superior semicircular canals, a superior semicircular canal dehiscence (SSCD). Over time, various conditions with a defect of the labyrinthine bony wall have been described in the literature (14–22). These are associated with a similar spectrum of symptoms and objective findings so that these conditions are now summarized under the general term of “third window syndrome” or “third window abnormalities” (23, 24). Wackym et al. (24) defined the following conditions associated with the term of third window syndrome: “SSCD, cochlea-facial nerve dehiscence, cochlea-internal carotid artery dehiscence, cochlea-internal auditory canal dehiscence, lateral semicircular canal-superior semicircular canal ampulla dehiscence, modiolus, perilymph fistula, posterior semicircular canal dehiscence, posterior semicircular canal-jugular bulb dehiscence, SSCD-subarcuate artery dehiscence, SSCD-superior petrosal vein dehiscence, vestibule-middle ear dehiscence, lateral semicircular canal-facial nerve dehiscence, wide vestibular aqueduct in children, post-traumatic hypermobile stapes footplate, otosclerosis with internal auditory canal involvement.” The objective findings of increased VEMP amplitudes and/or lower VEMP thresholds have been reported in patients with SSCD (25–28), posterior semicircular canal dehiscence (16, 29), large vestibular aqueduct (30), perilymph fistula (31), cochlea-facial nerve dehiscence (24), posterior semicircular canal-jugular bulb dehiscence (32), and SSCD-superior petrosal vein dehiscence (33). In these patients, the presence of a third window caused by an otic capsule defect changed the mechanical properties, i.e., the fluid dynamics, of the inner ear. It has been shown by measurements and models, that in SSCD ears incoming acoustic energy causes larger fluid displacement in the semicircular canals (34–36). Furthermore, animal studies demonstrated that this results in activation of semicircular canal neurons in addition to otolith neurons (37). These canal afferents project to the contralateral (external ocular) inferior oblique muscle as well as the ipsilateral sternocleidomastoid muscle (inhibitory) and thus their activity contributes to and enhances cVEMPs and oVEMPs (38).

Apart from defects in the bony labyrinth, other inner ear pathologies have the potential to impact inner ear mechanics. Endolymphatic hydrops for instance, which can have various causes (39, 40), is believed to have a huge impact on inner fluid mechanics (41–43). This argument is supported by VEMP studies in patients suffering from clinically diagnosed definite MD. Asymmetric, enhanced cVEMPs (44, 45) but also enhanced oVEMPs in MD patients have been reported (46, 47). These inner ear pathologies can therefore mimic third window syndrome with regard to VEMP test results. While ILS can mimic other cochleovestibular diseases in their symptoms and functional findings, it is unknown, if ILS can also mimic third window syndrome as was described for other inner ear pathologies.

The aim of this study was to review and describe oVEMPs and cVEMPs of patients with solely intracochlear localization of an ILS. By including only ICS patients, we sought to avoid those with direct impact of the intravestibular schwannoma on otolith organs which might lead to a change in VEMP results.

## Materials and Methods

### Study Design and Participants

In this retrospective analysis, patients of a single tertiary referral center were included. In a personal case series (SKP) at the University Hospital Halle of 53 consecutive patients with intralabyrinthine schwannoma (ILS), magnetic resonance images were analyzed for localization of the tumor. Patients with solely intracochlear schwannoma (ICS) in whom VEMP measurements had been performed between August 2015 and January 2020 were included in this study (ICS patient group). Patients without VEMP measurements or with other tumor localizations [see introduction according to Van Abel et al. (5)] were excluded from this study to avoid influence by direct impact of the tumor on the otoliths or by retrocochlear pathology.

Written informed consent was obtained from all patients. The study protocol was reviewed and approved by the responsible institutional review board (ethics committee of the Medical Faculty of Martin Luther University Halle-Wittenberg and the University Hospital Halle, approval number: 2019-26), and conducted according to the Declaration of Helsinki.

### VEMP Testing

The VEMP tests of the included patients were reviewed. All VEMP recordings were collected and analyzed using the Eclipse recording platform (Interacoustics A/S, Middelfart, Denmark). Self-adhesive Neuroline 720 surface electrodes (Ambu A/S, Ballerup, Denmark) were used for electromyogram (EMG) recording after the skin was prepared to provide impedances of 5 kΩ or less. For cVEMPs, the electrodes were placed over the middle of the sternocleidomastoid muscle ipsilateral to the stimulated ear and over the sternum. For oVEMP recordings, the electrodes were placed on the infra-orbital ridge 1 cm below the lower eyelid contralateral to the stimulated ear and about 2 cm below the first electrode. The ground electrode was always positioned on the forehead.

During VEMP testing the patients were sitting on a chair. They were asked to turn their head to the contralateral shoulder for cVEMP testing and hold this position to achieve a constant tonic activation of the sternocleidomastoid muscle (50–200 μV) during the whole recording period. During data acquisition the EMG was monitored and appropriate feedback was provided in real time to ensure that sufficient muscular contraction was sustained (48). For oVEMP testing, the patients were asked to keep their head in a neutral position and look up, maintaining an angle of 20–30°.

For both, cVEMP, and oVEMP testing, air-conducted 500 Hz tone bursts (1 cycle rise/fall time, 2 cycles plateau) were delivered by ER-3A insert earphones (3M, St. Paul, MS, USA) at 100 dB nHL.

The EMG signals were recorded in a −20 to 80 ms window relative to the onset of the stimulus. A bandpass filter of 10–1,000 Hz was applied and the artifact rejection level was set to 400 μV. The responses were averaged to at least 200 stimuli and at least two trials were recorded for each VEMP test.

### Specifying Tumor Location

All patients underwent MRI of the temporal bone with at least thin-sliced 3D T2-weighted and T1-weighetd images with contrast medium. In all included patients, the MRI was (retrospectively) systematically studied regarding the localization of the tumor. The classification suggested by Van Abel et al. (5) was used to classify tumor localization in the basal turn (BT), middle turn (MT), or apical turn (AT) of the cochlea, including combinations of these localizations. Additionally, localization of the tumor in the scala tympani (ST) and/or scala vestibuli (SV) was specified, if possible. The MRIs originated from different sources, often from outside our hospital, and thus, showed considerable differences in resolution. MRIs were not repeated, if the scans were sufficient for establishing the diagnosis of ICS.

### Exclusion of Third Window Syndromes

Temporal bone computed tomography (CT) scans or cone beam CTs were retrospectively analyzed for the presence of semicircular canal dehiscence, enlarged vestibular aqueduct, cochlea-facial nerve dehiscence, and other third window syndromes [see introduction and (24)]. It has to be noted, however, that the intention for performing the CT and cone beam CT scans were solely for preoperative evaluation of the bony anatomy prior to a possible surgery for tumor removal and hearing rehabilitation with a cochlear implant. They were performed after the diagnosis of an ILS was established by MRI and thus they usually did not include specific reconstructions for evaluation of “third windows” of the inner ear, e.g., no planes along the superior semicircular canal.

The patients' medical histories taken at initial presentation including audiological and vestibular complaints were retrospectively evaluated for typical symptoms of third window lesions including vertigo or oscillopsia induced by loud sounds/Tullio phenomenon, increased sensitivity to low frequency sounds, autophony, and pulsating tinnitus.

### Data Analysis

A VEMP was ultimately judged as present, when the putative response was clearly larger than the pre-stimulus waveforms, i.e., the background noise. The impact of muscle contraction on cVEMP results was reduced by averaging the root mean square of the EMG signal over the pre-stimulus window and for each recording frame to calculate the background EMG, i.e., the contraction strength.

The p13 n23 for cVEMPs and n10 p15 for oVEMPs were identified and peak latencies as well as peak-to-peak amplitudes were recorded. The p13 n23 peak-to-peak amplitude was normalized to the background EMG. The asymmetry ratio (AR) was calculated from the peak-to-peak amplitudes. In order to account for the side affected by the tumor (AS) and the non-affected side (NAS) and to overcome the drawback of absolute AR, a signed AR was used:

$$\begin{array}{l} AR\left(\%\right)=\frac{amplitude\;\left(AS\right)-amplitude\;\left(NAS\right)}{amplitude\;\left(AS\right)+amplitude\;\left(NAS\right)}*100. \end{array}$$

For cVEMPs, the AS refers to the response recorded from the ipsilateral sternocleidomastoid muscle and for oVEMPs the AS refers to the response recorded from the contralateral inferior oblique muscle. For cVEMPs, ARs above 30% or below −30% were considered abnormal (49). For oVEMPs, abnormal ARs were above 40% or below −40% (50, 51). Positive values of the AR indicate larger responses of the affected ear (enhanced), while negative values indicate smaller responses of the affected ear (reduced), respectively. If no response could be detected, the amplitude was set to 0 μV. For unilateral responses, the AR was therefore 100% or −100%.

VEMP analysis was performed by two blinded examiners. Normal distribution of the amplitude and latency data was confirmed by a Shapiro-Wilk test. The intraclass correlation coefficient [ICC (3, 1)] was calculated for the oVEMP and cVEMP ARs based on the analysis by the two examiners to assess the inter-rater reliability. If no responses could be detected on the AS and NAS, the AR was set to 0% for the statistical test (see following paragraph). Inter-rater agreement was considered “poor” for ICCs below 0.50, “moderate” between 0.50 and 0.75, “good” between 0.75 and 0.90, and “excellent” above 0.90 (52). Good or excellent agreement was considered acceptable for further analysis. The final latencies and amplitudes were the averages of the examiners. For absence of a response rated by one examiner but presence of a response rated by the other examiner, the amplitudes were the averages and the latencies were taken from the one examiner who rated the response to be present. The cVEMP and oVEMP latencies and amplitudes recorded from the AS were compared to the responses from the NAS as control by paired *t*-tests. A confidence level of 95% or above was considered to be significant (*p* < 0.05). SPSS statistics (IBM, Armonk, New York, USA) was used for all statistical analyses.

The VEMP results were related to tumor localization in a hypotheses generating descriptive analysis.

## Results

Twenty-six patients with solely intracochlear schwannoma (ICS) were identified. Six patients had not undergone VEMP testing and were therefore excluded. The analysis of the CTs or cone beam CTs (available in 16 patients) revealed a dehiscent superior semicircular canal in one case. This patient was excluded as well. There were no signs for other third mobile windows of the otic capsule. Thus, 19 patients with ICS were included in the study for final analyis. Of those, 10 patients were male, 9 were female. The mean age was 57.1 (SD: 13.4) years. In 8 patients, the left ear was affected, in 11 patients the tumor was located in the right ear. The mean hearing threshold [pure tone average at 0.5, 1, 2, and 4 kHz (4PTA)] was 90.8 (SD: 25.0) dB HL for the affected side (AS) and 18.7 (SD:13.3) dB HL for the non-affected side (NAS). Some of the patients reported pulsating tinnitus and very few patients reported autophony and increased sensitivity for low frequency sounds. Other typical clinical symptoms of third window lesions like oscillopsia or vertigo induced by loud sounds/Tullio phenomenon have not been observed in any of those patients.

Despite the different image resolution of the MRIs, it was possible to specify tumor localization according to basal turn (BT), middle turn (MT), and the apex (apical turn, AT) in all patients. Only in one patient, it was difficult to localize the tumor with respect to the scala tympani (ST), and/or scala vestibuli (SV). Data for all patients are summarized in Table 1. The tumor was located in the BT in 2 patients (11%), in the MT in 7 patients (37%), and in the AT in 1 patient (5%). In 4 patients (21%), the tumor was in the BT and MT, in 4 patients (21%) it was in the MT and AT, and in 1 patient (5%) it was in the BT, MT, and AT. With respect to the scalae, tumors were observed solely in ST in 5 patients (26%). In no patient, the tumor was solely located in SV, and in 14 patients (74%) it was located in both, ST, and SV.

**Table 1:** Demographic data, oVEMP and cVEMP asymmetry ratio (AR) results, and tumor localization of included patients.

**ID**	**Age range**	**AS**	**4PTA hearing level (dB)**		**VEMP AR (%)**		**ICS Location**	
			**AS**	**NAS**	**cVEMP**	**oVEMP**	**Cochlear turn**	**Scala**
1	45–50	R	75.00	16.25	n.a.	−2	(MT)+AT	ST+SV
2	45–50	R	>110.00	6.25	−87^*^	n.a.	(BT)+MT+AT	ST+SV
3	50–55	L	73.75	10.00	6	−29	(MT)+AT	ST+SV
4	30–31	L	97.50	2.50	−63^*^	n.a.	(BT)	ST+SV
5	65–70	R	80.00	28.75	−9	19	(BT)+MT	ST
6	70–75	L	>110.00	22.50	−65^*^	−100^*^	BT+(MT)	ST
7	75–80	L	>110.00	62.50	n.a.	n.a.	(MT)	ST+SV
8	55–60	R	>101.25	33.75	38^*^	7	MT	ST+SV
9	55–60	R	97.5	11.25	12	n.a.	AT	ST+SV
10	70–75	R	>110.00	18.75	−100^*^	−85^*^	MT	ST+SV
11	70–75	L	>110.00	23.75	−100^*^	n.a.	(MT) + (AT)	ST+SV
12	60–65	R	86.25	8.75	8	−100^*^	(MT)	ST
13	30–35	L	71.25	8.75	59^*^	76^*^	(MT)	ST+SV
14	50–55	R	67.50	13.75	38^*^	67^*^	(BT)+MT	ST+SV
15	60–65	R	>110.00	15.00	−16	−14	MT	ST
16	65–70	R	>110.00	10.00	n.a.	63^*^	MT+AT	ST+SV
17	60–65	L	91.25	21.25	12	−1	MT	ST
18	40–45	L	>110.00	23.75	n.a.	−100^*^	BT	ST+SV
19	55–60	R	92.50	17.50	33^*^	n.a.	BT+(MT)	ST+SV

4PTA, pure tone average at 0.5, 1, 3, 4 kHz; AS, affected side; NAS, non-affected side; AR, asymmetry ratio; ICS, intracochlear schwannoma; BT, basal turn; MT, middle turn; AT, apical turn; ST, scala tumpani; SV, scala vestibuli; (), partially;

* *abnormal AR*.

**Figure 1:**
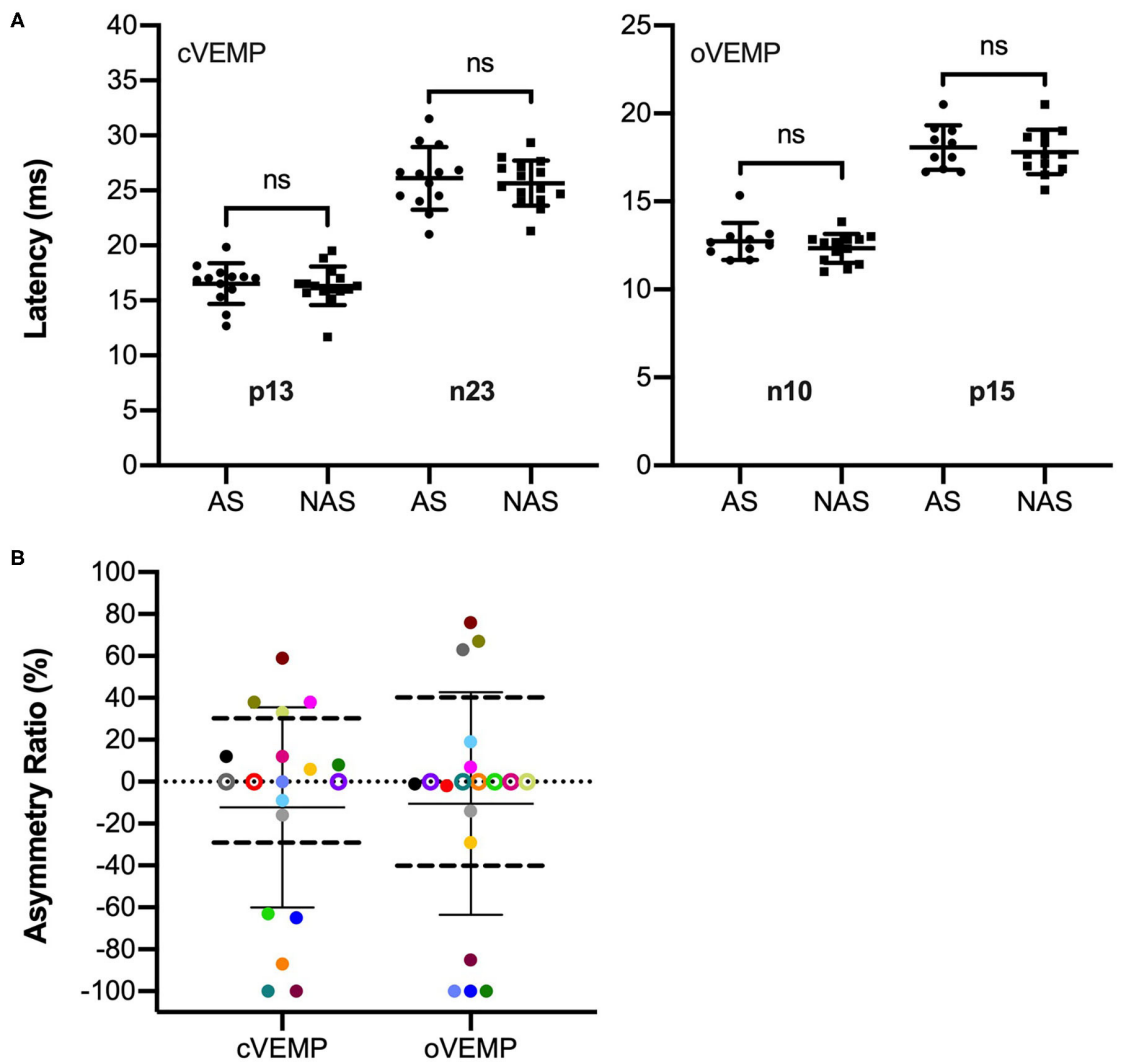
Latency and VEMP asymmetry ratio (AR) results of the included patients (*n* = 19). **(A)** Response latencies with means and standard deviations for the cVEMP p13 and n23 for the side affected by the tumor (AS) (*n* = 13) and the non-affected side (NAS) (*n* = 16) as well as oVEMP n10 and p15 latencies for the AS (*n* = 10) and NAS (*n* = 13). No significant differences were found between latencies of the AS and NAS. **(B)** Signed ARs for cVEMPs and oVEMPs with means and standard deviations. Patients are color coded as they contribute to both the oVEMP and cVEMP AR data. Negative values indicate larger responses on the NAS, positive values indicate larger responses on the AS. For cVEMPs, ARs exceeding ±30% were considered abnormal. For oVEMPs, abnormal ARs were larger/smaller than ±40%. The limits are illustrated by horizontal dashed lines. Data points above the thresholds represent enhanced VEMPs with respect to the AS, data points below the thresholds represent reduced VEMPs in the AS. Patients with absent responses on the AS are shown at AR = −100%. For patients with absent responses on both AS and NAS, the ARs are illustrated at AR = 0% as empty circles.

Regarding the VEMP analysis, the inter-rater reliability analysis by ICC revealed good to excellent agreement between the two raters. For cVEMPs, the single measure ICC was 0.990 with a 95% confidence interval from 0.996 to 0.975 [*F*_(18)_ = 205.248, *p* < 0.001]. For oVEMPs, the single measure ICC was 0.875 with a 95% confidence interval from 0.706 to 0.950 [*F*_(18)_ = 15.052, *p* < 0.001]. Cervical VEMPs could be recorded from the affected side (AS) in 13 cases (68%). In 4 cases, the response was absent in the non-affected side (NAS) as well. In the other 2 cases, the AS was the only ear without a response (AR = −100%). The oVEMP measurements showed responses of the AS in 10 patients (53%). In 6 cases, it was absent in both, AS and NAS. In 3 cases, the AS was the only side without a response (AR = −100%). The mean p13 n23 cVEMP amplitude was 0.46 (SD: 0.52) for the AS and 0.50 (SD: 0.45) for the NAS. For oVEMPs, the mean n10 p15 amplitude was 2.44 μV (SD: 4.27 μV) for the AS and 1.89 μV (SD: 2.10 μV) for the NAS. The mean cVEMP p13 latencies were 16.5 ms (SD: 1.8 ms) for the AS and 16.1 ms (SD: 1.7 ms) for the NAS, mean n23 latencies were 26.1 ms (SD: 2.9 ms) and 25.6 ms (SD: 2.2 ms), respectively. For oVEMPs, the mean n10 latencies were 12.7 ms (SD: 1.0 ms) for the AS and 12.4 ms (SD: 0.8 ms) for the NAS, and p15 latencies were 18.1 ms (SD: 1.3 ms) and 17.9 ms (SD: 1.2 ms), respectively. Between the AS and NAS, no significant difference was found for p13 and n23 cVEMP latencies [*t*_(12)_ = 1.267, *p* = 0.229; *t*_(12)_ = 1.216, *p* = 0.247] as well as for the n10 and p15 oVEMP latencies [*t*_(9)_ = 1.552, *p* = 0.155; *t*_(9)_ = 0.998, *p* = 0.344]. The results are illustrated in Figure 1A.

**Figure 2:**
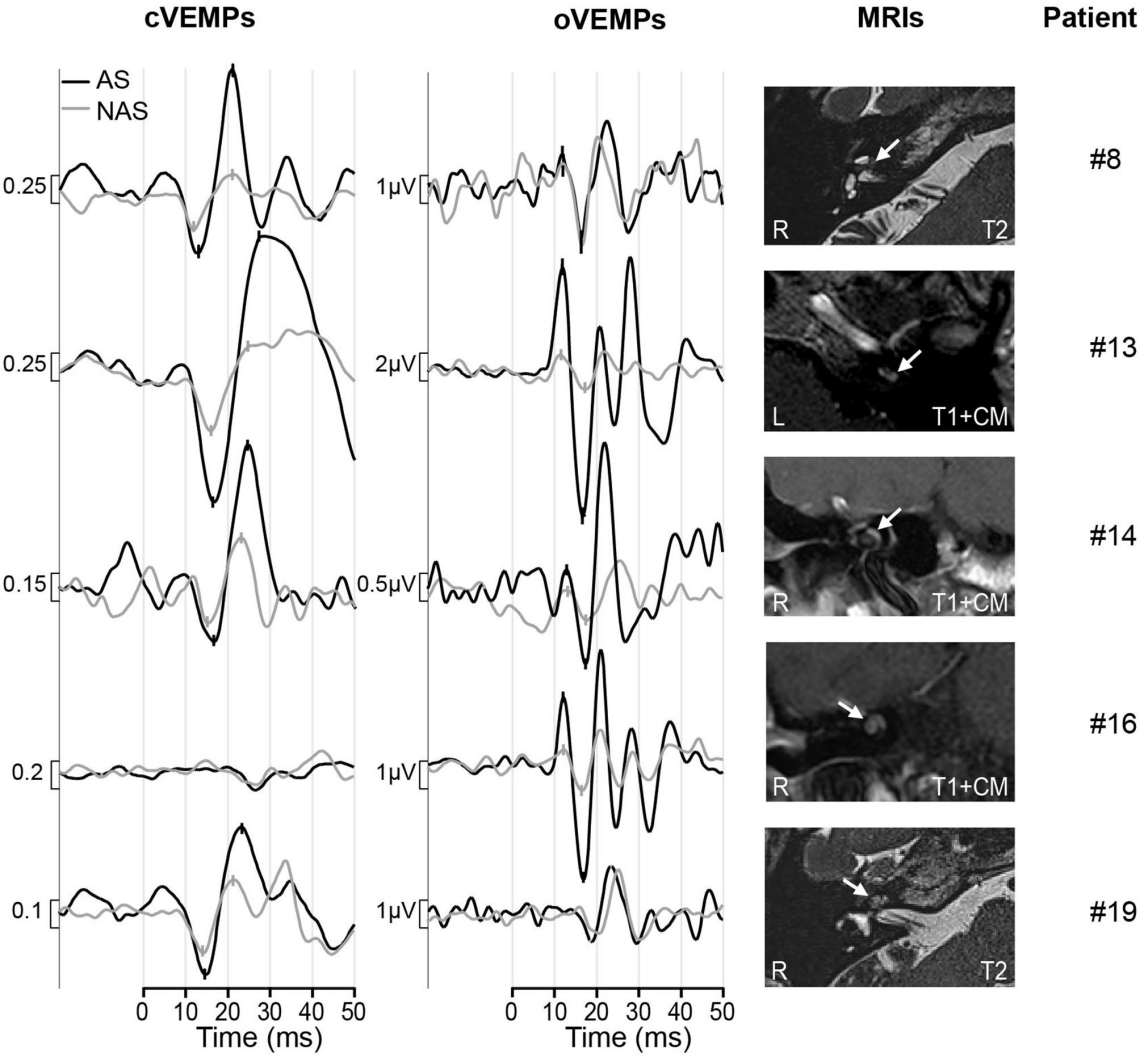
Results of patients with enhanced VEMPs. The black trace shows the response of the affected side (AS), the response from the non-affected side (NAS) is shown by the gray line. The p13, n23 peaks for cVEMPs and n10, p15 peaks for oVEMPs are marked. The MRI scans are shown in the right column with the tumors marked by white arrows. Patient #8, #13, #19: axial, Patient #14 and #16: coronal; R, right; L, left; T1+CM, t1-weighted with contrast medium; T2, t2-weighted.

The VEMP asymmetry ratio (AR) results are given for each patient in Table 1. Figure 1B illustrates the results in a boxplot. Patients are color coded as they contribute to both the oVEMP and cVEMP AR data. If no response could be recorded on both the AS and NAS, the AR was illustrated at 0% by an empty circle in the plot. The mean AR was −15.6% (SD: 53.6%) for cVEMPs and −15.3% (SD: 64.5%) for oVEMPs. For cVEMPs, the AR was smaller than −30%, i.e., asymmetrical with reduced responses on the AS, in 5 cases (26%), including the 2 cases with ARs of −100%. The AR was larger than 30%, i.e., asymmetrical with enhanced responses in the AS, in 4 cases (21%). Including the 3 cases with ARs of −100%, the oVEMP AR was smaller than −40% in 4 cases (21%) and larger than 40% in 3 cases (16%). In total, VEMPs were enhanced on the AS in 5 patients: in 2 patients only the cVEMPs (#8, #19), in 1 patient only the oVEMPs (#16), and in 2 patients both, the cVEMPs and oVEMPs (#13, #14). The VEMP results of these patients are illustrated in Figure 2.

CT or cone beam CT scans were available in four of five patients with enhanced VEMPs (patient #8 declined the CT) and did not show any signs of a third mobile window of the otic capsule.

The analysis of enhanced VEMPs and tumor localization showed that in 2 of the 5 patients with enhanced VEMPs only the MT (#8, #13), in 2 patients the BT and MT (#14, #19), and in 1 patient MT and AT (#16) was involved. A correlation of enhanced VEMPs and tumor localization with respect to the cochlear turn could therefore not be observed. With respect to the scala, the tumors were located in both, ST and SV, in all five patients with enhanced VEMPs and in none of the patients where only the ST was affected by the tumor.

## Discussion

Only a few studies reported VEMP results in patients with intralabyrinthine schwannoma (ILS) and mostly described absent or decreased cVEMPs and/or oVEMPs in these patients (6, 11, 12). The exact tumor localization and description of VEMP results in patients with solely intracochlear schwannomas (ICS) was done by Dubernard et al. (6) for 12 patients. Cervical VEMPs were absent or significantly reduced in 50% of the patients and “preserved” in the remaining 50%. Patients with intravestibular tumors were also examined in their study and were—not surprisingly—found to have a higher rate of absent or reduced cVEMPs, which is likely due to a direct impact of the tumor mass on the otolith organs. The results from our study are the first to systematically analyze both oVEMPs and cVEMPs in a series of cases with solely intracochlear tumors.

Despite the intracochlear position, the tumor affected the vestibular response which was found to be absent, reduced and in some cases enhanced. We observed absent or reduced cVEMPs in 47% of the patients and normal cVEMPs in 32%. This is in line with the results described by Dubernard et al. (6). The surprising result was that the cVEMPs were enhanced in 21% of the patients in our study. Ocular VEMPs were reduced or absent in 53% and normal in 32%. Enhanced oVEMPs were observed in 15% of the patients.

Many factors can cause reduced or absent VEMP responses. Particularly in central pathology, the VEMPs are absent, reduced and/or prolonged which can be an early indicator of pathology (53–56). Many studies reported reduced or absent VEMPs in patients with cochleovestibular schwannoma (i.e., vestibular schwannoma, see introduction) and reported a strong relationship with tumor size (57). Cervical VEMPs in 38 ears of Neurofibromatosis Type 2 patients with small cochleovestibular schwannomas were described by Holliday et al. (58). Normal results were found in 71% and abnormal cVEMPs were found in 29% of the patients with a correlation between abnormal cVEMPs and tumor size. VEMP asymmetry ratios (ARs) in patients with cochleovestibular schwannomas can also be used as a screening tool for assessing the function of the superior and inferior vestibular nerves before and after surgical intervention (10). VEMP abnormalities in these patients are attributed to compressional and neurotoxic effects on the nerve and reduced vascular supply of the labyrinth. However, retrocochlear pathology or direct impact of the tumor on the otoliths was excluded in our study by including only patients with solely intracochlear tumors. In addition, another patient with superior canal dehiscence was excluded, which could have acted as a confounding factor in the VEMP analysis. The pathophysiology of ICS leading to abnormal VEMPs is unknown and can only be speculated about. In patients with cochleovestibular schwannomas (without intracochlear localization of tumors), it has been reported that sensorineural hearing loss is associated with tumor-secreted factors containing pro-inflammatory cytokines which cause cochlear damage (59, 60). This could explain why large cochleovestibular schwannomas sometimes do not cause hearing loss while small ones do. This has not been investigated yet for ICS associated loss of otolith function but could be a similar mechanism. Possibly, the finding of reduced or absent VEMPs in these patients is attributed to a local cytotoxic effect conveyed by the labyrinthine fluids (6).

In the present study, the major and unexpected finding was that the VEMPs in ICS in some patients were enhanced but no latency prolongation was observed. Enhanced VEMPs are commonly seen in patients with third window syndrome. Thus, VEMPs have become a widely used tool in the diagnosis of third window syndrome (16, 24, 29–33) and are enhanced in those ears with a defect in the otic capsule (25–28). However, other conditions with endolymphatic hydrops such as Menière's disease have been shown to mimic third window syndrome showing reduced VEMP thresholds and enhanced amplitudes (44–47). To date, it was unknown that—with respect to VEMP results—other inner ear disorders, ICS in particular, have the potential to mimic third window syndrome as well. In our experience, management of patients with ILS is highly individual and detailed functional evaluation of the vestibular labyrinth is important for counseling these patients regarding treatment options (especially with respect to surgical tumor removal) and outcome predictions (61, 62).

To explain the cause of enhanced VEMP amplitudes in ICS patients, we assume that this is due to a change of inner ear (fluid) mechanics caused by the tumor. In this study, we only included ICS patients to avoid bias due to direct influence of the tumor on the otoliths (e.g., as in intravestibular or intravestibulocochlear schwannomas) or by tumor in the internal auditory canal. Measurements, models, and animal studies have shown that in patients with superior semicircular canal dehiscence, a third mobile window leads to larger fluid displacement in the semicircular canals which activates canal neurons contributing to the VEMP and enhancing it (34, 37, 63). This shows that mechanical changes cause enhanced VEMPs. It also supports the theory that mechanical changes can lead to enhanced VEMPs in patients with endolymphatic hydrops. The exact mechanisms of this observation are yet unknown. Endolymphatic hydrops can have various causes (39, 40). It seems possible that an obstructive tumor mass like an ICS has the potential to cause or act similar to endolymphatic hydrops. This idea is supported by the observation in our study that cVEMPs or oVEMPs were only enhanced, if both scala tympani (ST) and scala vestibuli (SV) were “blocked” by the tumor. This leads to the somehow contradictory observation that—with respect to VEMPs—a “third window syndrome” can act similar to a “minus 1 window syndrome” or “one window syndrome,” when the cochlear is “blocked” by a tumor.

In such situations, acoustic stimulation cannot lead to a fully developed traveling wave within the cochlea. Nevertheless, the stapes displacement must still be compensated by a reciprocal displacement of the round window membrane. It is conceivable that this may lead to a perilymph flow which is oscillating more or less directly between the oval window and the round window including a corresponding displacement of the basal basilar membrane. Clearly, in this configuration the fluid dynamics in the basal region of the cochlea would be significantly altered and—similar to the third window syndrome (36)—there would be higher flow velocities close to the saccule which may lead to enhanced VEMPs. It appears also possible that the cochlear blockage leads to a suppression of the piston-like stapes motion (PSM) and that the acoustic stimulation leads only to a “rocking stapes motion” (RSM) which does not create any net fluid displacement within the otic capsule. It has been shown [Figure 5 in Edom et al. (64)] that RSM leads to significantly increased perilymph flow in the basal region of the cochlea which may affect saccular stimulation and be connected to enhanced VEMPs. Computer-modeling of the fluid dynamics of a “blocked” cochlea may have the potential to give answers to the question if the mass effect without concomitant neural damage could cause the enhancement of VEMPs and should be considered in future studies.

Limitations of the study include its retrospective design, which is due to the nature of the observation which was more or less accidental. This is also a reason, why the study did not include threshold measurements. Since VEMPs evoked by bone conducted vibration were not available at that time, only air conduction was used for stimulation. While any third window abnormalities were excluded in most patients, CT scans were not available in 1 of the 5 patients with enhanced VEMPs, and CTs (although thin-sliced) were technically not targeted specifically on exclusion of bony defects of the inner ear. These limitations can be addressed in further studies including specific history taking (i.e., checklists for symptoms of third window syndromes), threshold measurements in all patients with enhanced VEMPs as well as specific CT scans in these patients. Another aspect which has to be considered in further studies is the evolution of VEMPs in these patients. It should be examined, how the VEMP amplitudes and latencies change over time, possibly in the course of tumor growth. Regarding the different outcomes, i.e., especially reduced or absent in contrast to normal or enhanced VEMPs, the tumors' intrinsic biology with respect to tumor secreted factors should be investigated as was done for cochleovestibular schwannomas causing hearing loss (59, 60). This is important to assess the clinical relevance of normal, absent or reduced, and enhanced VEMPs and might become beneficial for counseling ICS patients.

## Conclusion

We described that enhanced VEMP amplitudes could be observed in patients with intracochlear schwannoma. It was an unexpected novelty that in addition to conditions described by the general term of the third window syndrome, or in Menière's disease, VEMP amplitudes can be enhanced in patients with intracochlear schwannoma. Response latencies were not significantly different between the side affected by the tumor and the non-affected side. Intracochlear tumors should therefore be added to the list of conditions which may cause increased VEMP amplitudes. Since management of patients with intracochlear schwannomas is highly individual, these findings might become beneficial for counseling these patients regarding treatment options and outcome predictions.

## Data Availability Statement

The raw data supporting the conclusions of this article will be made available by the authors, without undue reservation.

## Ethics Statement

The studies involving human participants were reviewed and approved by Ethics committee of the Medical Faculty of Martin Luther University Halle-Wittenberg and the University Hospital Halle. The patients/participants provided their written informed consent to participate in this study.

## Author Contributions

LF and SP contributed the conception and design of the study and organized the database. LF, SP, and SK performed the data analysis. TR contributed to the data analysis and the visualization of results. IC and DO made contributions with interpretation of the results and generation of hypotheses. LF wrote the first draft of the manuscript. All authors contributed to the manuscript revision, read, and approved the submitted version.

## Conflict of Interest

The authors declare that the research was conducted in the absence of any commercial or financial relationships that could be construed as a potential conflict of interest.

## Abbreviations

ILS: intralabyrinthine schwannoma
MD: Menière's disease
MRI: magnetic resonance imaging
ICS: intracochlear schwannoma
cVEMP: cervical vestibular evoked myogenic potential
oVEMP: ocular vestibular evoked myogenic potential
SSCD: superior semicircular canal dehiscence
VEMP: vestibular evoked myogenic potential
EMG: electromyogram
BT: basal turn
MT: middle turn
AT: apical turn
ST: scala tympani
SV: scala vestibuli
CT: computed tomography
AR: asymmetry ratio
AS: affected side
NAS: non-affected side
ICC: intraclass correlation coefficient.
